# Pedagogical Efficacy of LLM-Generated Synthetic Data Versus Real-World Clinical Records: A Randomized Controlled Non-Inferiority Trial

**DOI:** 10.5334/pme.2535

**Published:** 2026-09-09

**Authors:** Zhongkun Zuo, Jianyu Fang, Muyao Ye, Zedong Li

**Affiliations:** 1 Department of Thyroid Surgery, The Second Xiangya Hospital, Central South University, No. 139 Renmin Middle Road, Changsha 410011, Hunan, China; 2 Department of General Surgery, The Second Xiangya Hospital, Central South University, Changsha, Hunan, China; 3 Department of Nursing, The Second Xiangya Hospital, Central South University, Changsha, Hunan, China; 4 Department of Nephrology, The Second Xiangya Hospital, Central South University, Changsha, Hunan, China; 5 Department of General Surgery, Xiangya Hospital, Central South University, Changsha, Hunan, China; 6 Department of Gastrointestinal Surgery, Xiangya Hospital, Central South University, Changsha, Hunan, China; 7 National Clinical Research Center for Geriatric Disorders, Xiangya Hospital, Central South University, Changsha, Hunan, China

## Abstract

**Background:**

Expert-reviewed clinical cases generated by large language models (LLMs) may supplement case resources in medical education, but their short-term educational performance relative to real-case-derived teaching materials remains uncertain. We compared immediate post-training test performance after teaching with the two types of case materials and assessed non-inferiority against a prespecified margin.

**Methods:**

We conducted a prospective, parallel-group, randomized non-inferiority trial. Through the Wenjuanxing online platform, participants were randomized 1:1 to learn with either real-case-derived teaching cases compiled by clinicians and reviewed by experts or AI-generated clinical cases produced by Gemini 3.0 Pro from fully de-identified matched real cases and reviewed by three senior general surgery specialists with full-professor rank. The primary outcome was the total score on an independent 10-item immediate post-training test (0–10 points), with a prespecified non-inferiority margin of –0.5 points. Secondary outcomes included the training-phase performance score, learning efficiency index, single-item mental effort rating, case realism, and case-source judgment.

**Results:**

A total of 403 participants were randomized, of whom 386 were included in the modified intention-to-treat analysis: 192 in the real-case group and 194 in the AI-generated case group. The mean post-training test score was 4.95 (SD, 3.35) in the real-case group and 4.61 (SD, 3.35) in the AI-generated case group. The mean difference (AI-generated minus real-case group) was –0.335 points (95% CI, –1.006 to 0.337). Because the lower bound of the confidence interval was below the prespecified non-inferiority margin of –0.5 points, non-inferiority was not demonstrated (one-sided P = 0.314). No significant between-group differences were observed in the training-phase performance score, learning efficiency index, or single-item mental effort rating. AI-generated cases received lower realism ratings for Level 3 cases. The proportion of participants with at least one high-confidence completely incorrect response was 1.6% in the real-case group and 2.1% in the AI-generated case group.

**Conclusions:**

In this short-term, text-based online case-learning setting, no statistically significant between-group difference was observed in immediate post-training test performance; however, non-inferiority of AI-generated clinical cases relative to real-case-derived teaching materials was not demonstrated.

## 1. Introduction

Clinical reasoning is central to clinical competence and is a key goal of medical education. It encompasses multiple cognitive processes, including information gathering, problem representation, hypothesis generation, weighing of evidence, and diagnostic and management decisions [1,2]. By immersing learners in varied patient scenarios, case-based teaching has long been used to teach this skill and to connect classroom knowledge with clinical practice [3]. Its effectiveness, however, depends heavily on the quality, diversity, and authenticity of the case materials [4]. Although real cases can convey the complexity and uncertainty of clinical practice, their use in teaching is constrained by patient privacy, de-identification requirements, data governance, and the costs of case acquisition and standardization [5,6,7]. Securing a sufficient supply of authentic teaching cases in a sustainable and controllable way therefore remains a recurring practical constraint for medical educators.

The emergence of large language models (LLMs) offers one possible way to address the supply of teaching cases. [8]. In collaboration with clinical teachers, these models can rapidly generate structured, realistic, and editable case text aligned with defined learning objectives, supporting the standardization, updating, and large-scale production of teaching materials [9,10,11]. Generating cases from de-identified inputs may also reduce learners’ direct exposure to original patient records [12] and improve the flexibility and accessibility of educational activities [13]. This expectation, however, has outpaced the evidence. Such teaching cases still derive from real patient information and should not be treated as synthetic data fully independent of real data; generative models may also memorize and reproduce training data, so privacy and re-identification risks are not eliminated by generation alone [14]. Generated content may contain factual errors, illogical reasoning, or insufficient clinical cues, and may be especially limited in reproducing the ambiguity and uncertainty of complex cases. Expert review is therefore necessary before educational use [9,15]. Despite considerable enthusiasm, randomized studies directly comparing the educational effects of expert-reviewed AI-generated clinical cases with matched real cases remain scarce. Current evidence is insufficient to determine whether such cases produce measurable learning gains, let alone whether they can replace real cases [16,17].

This evidence gap has practical consequences. If educators assume, without evidence, that AI-generated and real cases are equivalent and substitute one for the other, they may compromise learning quality, case authenticity, and possibly patient safety. Conversely, rejecting AI-generated cases outright may forgo their potential value for scaling case production and reducing privacy exposure. A prudent choice between these positions requires direct comparative evidence. We conducted a prospective, parallel-group, randomized non-inferiority educational trial. AI-generated clinical cases were produced from matched, fully de-identified real cases, reviewed by three senior general surgery experts, and compared with real-case-derived teaching materials from the same source. We hypothesized a priori that, against a prespecified non-inferiority margin, the AI-generated case group would be non-inferior to the real-case group in immediate post-training test performance.

## 2. Materials and Methods

### 2.1 Study Design and Participants

This prospective, parallel-group, randomized controlled educational study used a non-inferiority design to compare the immediate post-training test performance of medical learners after learning with expert-reviewed AI-generated clinical cases or real-case-derived teaching materials. The study was reported with reference to CONSORT items applicable to parallel-group randomized non-inferiority trials.

The study was conducted online from November to December 2025, with recruitment links distributed through targeted WeChat groups. Eligible participants were medical learners at the training stages covered by this study, including medical students in their third year or above and resident physicians. Participants were required to have stable internet access and to provide electronic informed consent. The study was approved by the Ethics Committee of the Second Xiangya Hospital, Central South University (approval no. LYEC2025-0334). All participants provided electronic informed consent before enrollment.

### 2.2 Randomization, Masking, and Analysis Set

Participants were automatically assigned in a 1:1 ratio to the real-case group or the AI-generated case group by the built-in randomization system of the Wenjuanxing platform, without stratification or block randomization. Investigators could not view, predict, or modify the next allocation before assignment. Investigators responsible for case-material preparation and platform setup were aware of group assignment, whereas participants were not informed of their assignment or of the source of the cases they read. Data-cleaning and statistical-analysis personnel were masked to group identity while processing the data.

After questionnaire completion, records were excluded if the total response time was shorter than 5 minutes or if responses showed a completely uniform pattern across the relevant items. The modified intention-to-treat (mITT) set was defined as randomized participants who met these data-quality criteria and was analyzed according to original randomized assignment. The numbers of participants randomized, excluded, and included in the final analysis set are reported in the Results.

### 2.3 Preparation and Review of Case Materials

Materials for the real-case group were compiled by experienced clinicians from fully de-identified real cases in general surgery into structured teaching cases and were reviewed by clinical experts. AI-generated clinical cases were produced with Gemini 3.0 Pro using matched, fully de-identified real cases as input; the model was accessed through Google AI Studio. Direct identifiers were removed before input to the model, while the clinical information needed to construct a complete teaching case was retained. Generation used the default run settings of Google AI Studio, with no manual modification of sampling parameters and no fixed random seed. The model was instructed to generate structured teaching cases while preserving the target diagnosis, key clinical cues, clinical logic, and complexity level of the matched real cases. Model outputs were not manually edited; cases that did not meet the review criteria were regenerated.

Three senior general surgery specialists with full-professor rank independently reviewed each pair of real-case-derived material and AI-generated clinical case. The review focused on the target diagnosis, core learning objectives, key clinical cues, case complexity, required diagnostic and management decisions, clinical logic, and educational suitability. Materials entered the formal study only after all three experts unanimously agreed that the case pair was matched and adequate. The full text of the five AI-generated clinical cases is provided in Supplementary Material S1; the real-case-derived materials are not disclosed because of patient-privacy and data-governance requirements.

Before the study, the five case pairs were classified into three levels of educational complexity and reviewed by clinical experts. Level 1 comprised cases with typical presentations, a single principal diagnosis, and a direct management pathway. Level 2 required integration of multiple clinical findings, consideration of differential diagnoses or common complications, and multistep decision-making. Level 3 involved acute critical illness, severe complications, hemodynamic instability, or multisystem involvement requiring prioritized resuscitation and emergency intervention. Case 1 was Level 1, Cases 2 and 3 were Level 2, and Cases 4 and 5 were Level 3. Each pair of real-case-derived material and AI-generated clinical case shared the same complexity level. No separate inter-rater agreement statistic, such as kappa or ICC, was calculated for this expert-consensus process.

### 2.4 Study Procedure and Outcome Measures

The online study comprised four consecutive phases. The first phase collected participants’ training stage, sex, prior rotation experience, baseline clinical confidence, and learning preferences.

The second phase was a five-case training phase. Participants completed five cases in sequence, each containing one single-best-answer multiple-choice item for diagnosis, reasoning, and decision-making. Each item scored 1 point if correct and 0 if incorrect, yielding a per-case score from 0 to 3. Across the five cases, these scores formed the training-phase performance score, ranging from 0 to 15. After submitting answers to the three items for each case, participants successively rated their confidence in their answers for that case (1–4), case realism (1–5), and perceived difficulty (1–3), and judged the case source as real, AI-generated, or uncertain. The platform displayed the reference answers and a brief explanation for each case only after these ratings were completed. Reading time and answering time were recorded separately for each case.

The third phase was an independent 10-item immediate post-training test. Both groups completed the same items, scored 1 point for a correct answer and 0 for an incorrect answer, yielding a total score from 0 to 10. The test was closed book, and participants were instructed not to review the training cases or use external resources. The total post-training test score was the primary outcome and represented immediate post-training test performance under the study conditions. Because no pre-training knowledge test or delayed follow-up test was administered, this outcome was not interpreted as learning gain or knowledge retention. A score of 6 or higher was defined as passing and served as a descriptive secondary outcome.

The fourth phase was a post-task questionnaire. Using a single 1–9 item, participants rated the overall mental effort required to complete the case training and testing; this was a single-item subjective mental-effort rating and not the full NASA-TLX. Participants also reported overall satisfaction (1–5) and attitudes toward AI-assisted learning. The case-source judgment task was a descriptive task designed for this study and was not a previously validated measurement instrument.

All training-phase and post-training test items were reviewed by three senior general surgery specialists with full-professor rank to evaluate clinical accuracy, content relevance, and the reasonableness of the answer keys. No formal pilot testing, quantitative content-validity index assessment, or formal Bloom cognitive-level classification was performed. Accordingly, diagnosis, reasoning, and decision-making were treated only as item-type labels rather than validated subscales of clinical ability. All multiple-choice items were scored automatically by the Wenjuanxing platform using the prespecified answer keys, with no human raters involved. The full questionnaire, items, options, answer keys, and scoring rules are provided in Supplementary Material S1; outcome definitions, measurement time points, and scoring ranges are provided in Supplementary Table S1.

Secondary learning outcomes included the training-phase performance score and the learning efficiency index, defined as the training-phase performance score divided by the cumulative reading time for the five cases (in minutes). Case realism, case-source judgment, mental effort, satisfaction, and attitudes toward AI were treated as secondary or descriptive outcomes. Analyses of the training-phase diagnosis, reasoning, and decision-making item groups, analyses stratified by training stage and case complexity, and the high-confidence completely incorrect response were treated as exploratory. A high-confidence completely incorrect response was defined post hoc as a per-case score of 0/3 with a case-level confidence rating of 4/4; the participant-level outcome was the occurrence of at least one such event across the five cases.

### 2.5 Sample Size and Statistical Analysis

At the design stage, a difference of –0.5 points in the mean 10-item post-training test score (AI-generated minus real-case group) was set as an exploratory non-inferiority margin, equivalent to 5% of the total score. This margin was smaller than the value of a single item. Based on the expected standard deviation of 1.5 points used for sample-size planning, the margin corresponded to approximately 0.33 standard deviations. In setting this margin, the study team also considered the potential advantages of AI-generated clinical cases for case expansion, content updating, and reducing privacy exposure to real cases. The margin was not a validated minimal educationally important difference. Assuming a true between-group mean difference of 0, a one-sided alpha of 0.025, and 80% power, the minimum sample size was 142 participants per group.

The primary analysis was conducted in the mITT set. The primary estimand was the difference in mean post-training test score (AI-generated minus real-case group). The mean difference and its two-sided 95% confidence interval were estimated using Welch’s two-sample method. Non-inferiority was concluded only if the lower bound of the confidence interval exceeded –0.5 points, and the corresponding one-sided non-inferiority P value was also reported. The two-sided between-group difference test was reported separately and was not used to determine non-inferiority.

The training-phase performance score was compared using the Wilcoxon rank-sum test. The learning efficiency index and mental effort ratings were compared using Welch’s two-sample test. The pass rate was compared using the Yates-corrected chi-square test, and the categorical distributions of satisfaction and attitudes toward AI were compared using the Pearson chi-square test. Case-source judgments were summarized descriptively by group. When realism, reading time, and case scores were analyzed by case complexity, participant-clustered robust standard errors were used to account for repeated case observations within participants.

Analyses of the training-phase diagnosis, reasoning, and decision-making item groups used the participant as the unit of analysis. For each participant, the mean proportion correct across the five items in each category was first calculated, and between-group differences were then compared using Welch’s two-sample test, with Holm correction for the three comparisons. The high-confidence completely incorrect response was compared using Fisher’s exact test on participant-level event proportions, with the risk difference and its 95% confidence interval reported.

Cronbach’s alpha and bootstrap 95% confidence intervals based on 5000 resamples were calculated separately for the 15-item training-phase assessment and the 10-item post-training test. Item difficulty was defined as the proportion correct, and corrected item-total correlations were calculated; upper-lower discrimination indices were additionally calculated for the training-phase items. These analyses were used only to evaluate reliability and item performance in the present sample and were not interpreted as complete construct-validity evidence. Except for the one-sided non-inferiority test, all statistical tests were two-sided, with P < 0.05 considered statistically significant. Analyses were performed with R 4.4.3.

## 3. Results

### 3.1 Participants and Assessments

**Figure 1:**
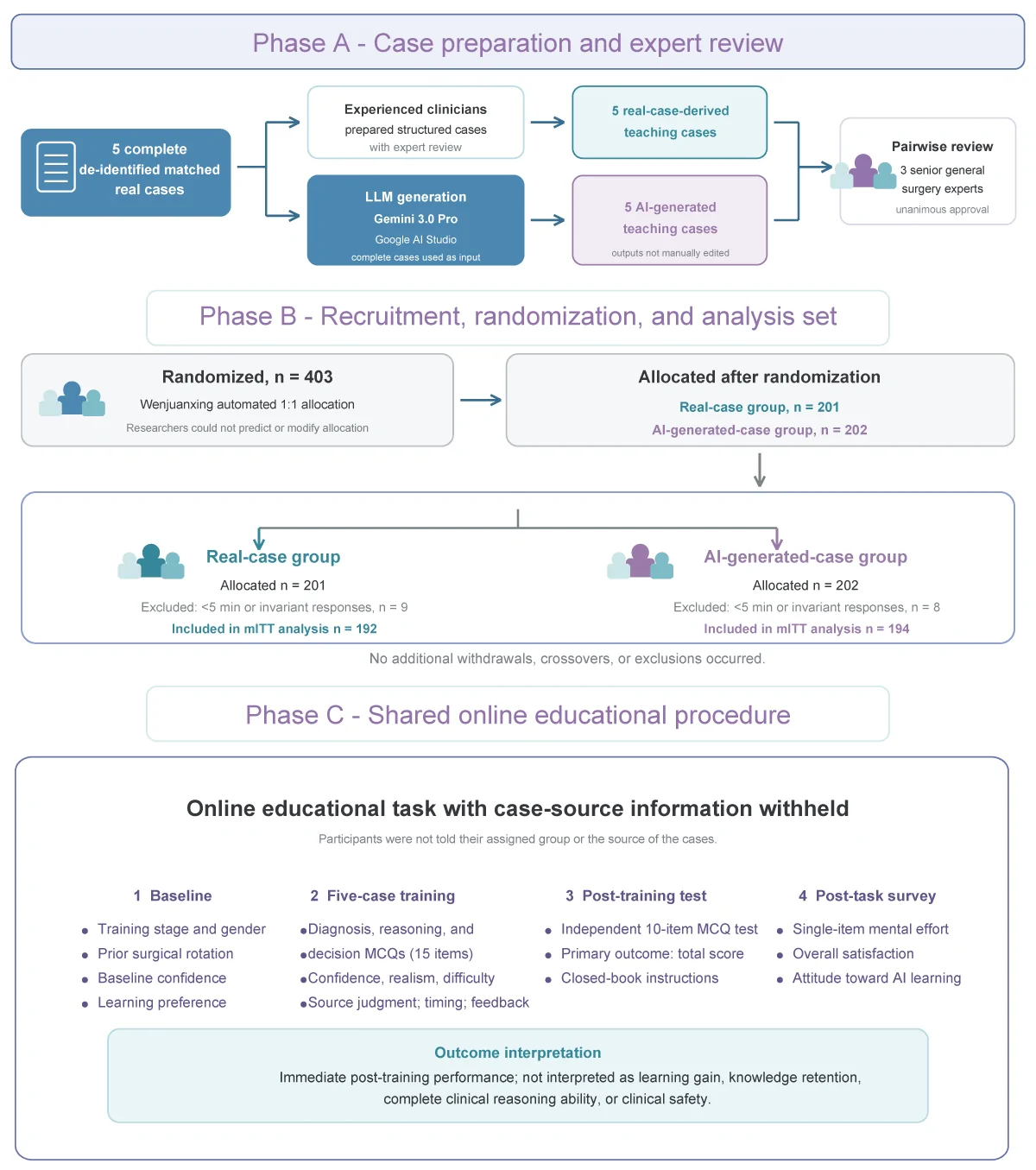
Preparation of study materials, participant flow, and the shared online educational process. Phase A shows how five fully de-identified, matched real cases served as the common source for both groups’ materials: experienced clinicians compiled them into structured real-case-derived teaching cases, while matched AI-generated teaching cases were produced using Gemini 3.0 Pro through Google AI Studio, with model outputs not manually edited. Each case pair entered the study only after unanimous review by three senior general surgery specialists with full-professor rank. Phase B shows automatic 1:1 randomization on the Wenjuanxing platform and the modified intention-to-treat set. After randomization, 9 participants in the real-case group and 8 in the AI-generated case group were excluded because the total response time was shorter than 5 minutes or because responses showed a completely uniform pattern; there were no other withdrawals, group changes, or exclusions. Phase C outlines the shared online educational process. Participants were not informed of their group or of the case source; this statement does not imply that case-source masking was necessarily successful.

A total of 403 participants were randomized to the real-case group (n = 201) or the AI-generated case group (n = 202). After randomization, 9 participants in the real-case group and 8 in the AI-generated case group were excluded for failing to meet the questionnaire data-quality criteria. The mITT analysis ultimately included 192 and 194 participants, respectively (Figure 1). Baseline characteristics of the two groups are shown in Table 1. The groups had similar distributions of training stage, sex, prior rotation experience, baseline confidence, and learning preferences (all P > 0.05).

**Table 1:** Baseline characteristics.


CHARACTERISTIC	REAL CASES N = 192	AI-GENERATED CASES N = 194	P VALUE

Training stage			0.150

Year 3	48 (25.0%)	67 (34.5%)	

Year 4	67 (34.9%)	53 (27.3%)	

Intern	42 (21.9%)	36 (18.6%)	

Resident	35 (18.2%)	38 (19.6%)	

Male gender	96 (50.0%)	99 (51.0%)	0.800

Prior surgical rotation	99 (51.6%)	85 (43.8%)	0.130

Baseline confidence			0.300

1	3 (1.6%)	1 (0.5%)	

2	33 (17.2%)	31 (16.0%)	

3	93 (48.4%)	82 (42.3%)	

4	54 (28.1%)	62 (32.0%)	

5	9 (4.7%)	18 (9.3%)	

Learning preference			0.900

Preference 1	57 (29.7%)	58 (29.9%)	

Preference 2	70 (36.5%)	75 (38.7%)	

Preference 3	65 (33.9%)	61 (31.4%)	


Values are n (%). P values are from Pearson chi-square or Fisher exact tests, as appropriate.

For the 15-item training-phase assessment, Cronbach’s alpha was 0.913 (bootstrap 95% CI, 0.901 to 0.924), item difficulty indices ranged from 0.298 to 0.676, and corrected item-total correlations ranged from 0.458 to 0.703 (Table 2). For the 10-item post-training test, Cronbach’s alpha was 0.879 (bootstrap 95% CI, 0.861 to 0.895; Supplementary Table S2).

**Table 2:** Item analysis of the 15-item training-phase assessment.


ITEM	DIFFICULTY INDEX	CORRECTED ITEM-TOTAL CORRELATION	UPPER-LOWER DISCRIMINATION	ALPHA IF ITEM DELETED

Case 1: diagnosis	0.676	0.595	0.740	0.908

Case 1: reasoning	0.630	0.608	0.772	0.908

Case 1: decision-making	0.609	0.578	0.772	0.909

Case 2: diagnosis	0.549	0.657	0.853	0.906

Case 2: reasoning	0.495	0.610	0.778	0.907

Case 2: decision-making	0.394	0.556	0.718	0.909

Case 3: diagnosis	0.557	0.684	0.902	0.905

Case 3: reasoning	0.523	0.650	0.827	0.906

Case 3: decision-making	0.438	0.690	0.875	0.905

Case 4: diagnosis	0.427	0.689	0.909	0.905

Case 4: reasoning	0.373	0.579	0.726	0.908

Case 4: decision-making	0.298	0.471	0.544	0.912

Case 5: diagnosis	0.438	0.703	0.901	0.904

Case 5: reasoning	0.368	0.630	0.784	0.907

Case 5: decision-making	0.298	0.458	0.527	0.912


Overall Cronbach alpha = 0.913 (bootstrap 95% CI 0.901 to 0.924). Difficulty index is the proportion correct; higher values indicate easier items.

### 3.2 Primary Outcome

The mean post-training test score was 4.95 (SD, 3.35) in the real-case group and 4.61 (SD, 3.35) in the AI-generated case group. The mean difference (AI-generated minus real-case group) was –0.335 points (95% CI, –1.006 to 0.337). Because the lower bound of the confidence interval was below the prespecified non-inferiority margin of –0.5 points, non-inferiority was not demonstrated (one-sided P = 0.314; Table 3; Supplementary Figure S1). A total of 82 of 192 participants (42.7%) in the real-case group and 77 of 194 participants (39.7%) in the AI-generated case group passed the post-training test (P = 0.618; Table 3).

**Table 3:** Learning outcomes.


OUTCOME	REAL CASES N = 192	AI-GENERATED CASES N = 194	EFFECT ESTIMATE (AI – REAL)	P VALUE

Training-phase score (0–15)	7.27 (4.97)	6.88 (4.85)	–0.39	0.419

Post-training score (0–10)	4.95 (3.35)	4.61 (3.35)	–0.335 (–1.006 to 0.337)	0.314

Pass rate (score >= 6)	82/192 (42.7%)	77/194 (39.7%)	–3.0 percentage points	0.618


Values are mean (SD) unless otherwise stated. For the primary post-training outcome, the prespecified non-inferiority margin was –0.5 points; the confidence interval crossed this margin, so non-inferiority was not demonstrated. P = 0.314 is the one-sided non-inferiority P value. Training-phase score used the Wilcoxon rank-sum test; pass rate used the Yates-corrected chi-square test.

### 3.3 Secondary Learning Outcomes and Learner Experience

**Figure 2:**
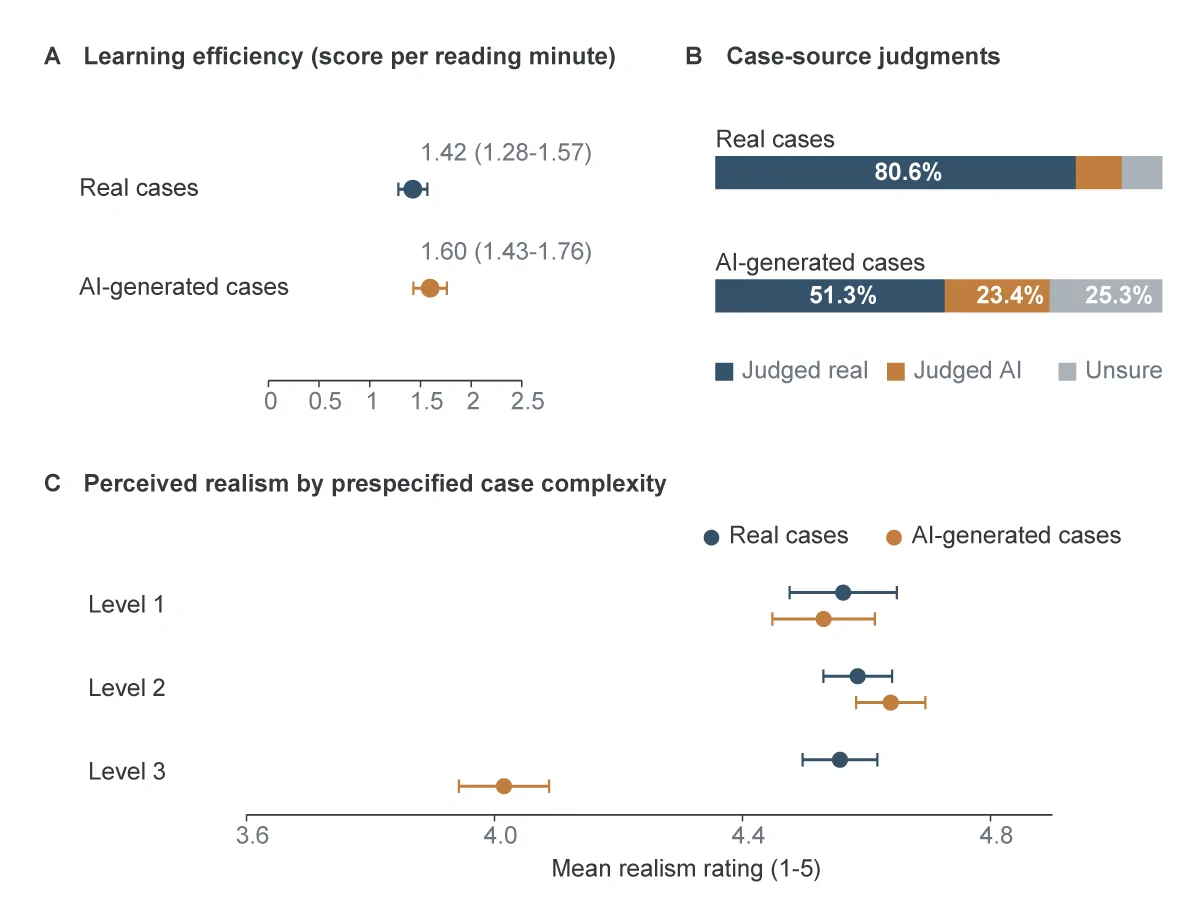
Learning process and case perceptions. **(A)** Learning efficiency index, defined as the training-phase performance score divided by cumulative reading time for the five cases (in minutes). **(B)** Distribution of case-source judgments; each participant made five judgments, and percentages were calculated by randomized group. **(C)** Mean case realism ratings summarized by prespecified case-complexity level. Case 1 was Level 1, Cases 2 and 3 were Level 2, and Cases 4 and 5 were Level 3. Error bars indicate descriptive 95% confidence intervals.

The training-phase performance score was 7.27 (SD, 4.97) in the real-case group and 6.88 (SD, 4.85) in the AI-generated case group (P = 0.419; Table 3). The learning efficiency index was 1.42 points per reading minute (SD, 1.01) and 1.60 points per reading minute (SD, 1.17), respectively (P = 0.126; Figure 2A).

The single-item mental effort rating was 4.79 (SD, 1.41) in the real-case group and 4.74 (SD, 1.44) in the AI-generated case group, with a mean difference of –0.05 points (95% CI, –0.34 to 0.23; P = 0.707). No significant between-group differences were observed in overall satisfaction (P = 0.490) or in the distribution of attitudes toward AI (P = 0.521; Table 4).

**Table 4:** Learner experience and attitudes.


OUTCOME	REAL CASES N = 192	AI-GENERATED CASES N = 194	EFFECT OR COMPARISON	P VALUE

Perceived mental effort (1–9)	4.79 (1.41)	4.74 (1.44)	–0.05 (–0.34 to 0.23)	0.707

Overall satisfaction (1–5)	4.22 (0.72)	4.30 (0.73)	Categorical distribution	0.490

AI attitude: support	113 (58.9%)	115 (59.3%)		

AI attitude: neutral	58 (30.2%)	64 (33.0%)	Three-category distribution	0.521

AI attitude: oppose	21 (10.9%)	15 (7.7%)		


Mental effort was measured with a single-item 1–9 scale, not the NASA-TLX. The mental-effort comparison used Welch’s t test; satisfaction and AI attitude used categorical chi-square tests.

### 3.4 Case Perceptions and Exploratory Analyses

Among the 960 case-source judgments in the real-case group, 774 (80.6%) were judged as real cases, 99 (10.3%) as AI-generated cases, and 87 (9.1%) as uncertain. Among the 970 judgments in the AI-generated case group, the corresponding numbers were 498 (51.3%), 227 (23.4%), and 245 (25.3%), respectively (Figure 2B).

No significant between-group differences in case realism ratings were observed for Level 1 or Level 2 cases. For Level 3 cases, AI-generated cases received lower realism ratings than real cases, with a mean difference of –0.542 points (95% CI, –0.632 to –0.451; P < 0.001; Figure 2C; Supplementary Table S3). For Level 3 cases, the training-phase case score was also lower for AI-generated cases than for real cases (mean difference, –0.21 points; 95% CI, –0.41 to –0.01; P = 0.038; Supplementary Table S3).

An indirect comparability analysis of the case materials showed that the cumulative reading time for AI-generated cases was significantly shorter than that for real cases (adjusted mean difference, –9.17 seconds; 95% CI, –9.90 to –8.45; P < 0.001), and this difference was consistent across the three complexity levels (Supplementary Tables S3 and S4). No significant between-group differences were observed in answering time (0.75 seconds, P = 0.123), perceived difficulty (–0.01, P = 0.463), or training-phase case score (–0.08, P = 0.442). The overall case realism rating of AI-generated cases was slightly lower (–0.20; 95% CI, –0.26 to –0.15; P < 0.001; Supplementary Table S4).

**Figure 3:**
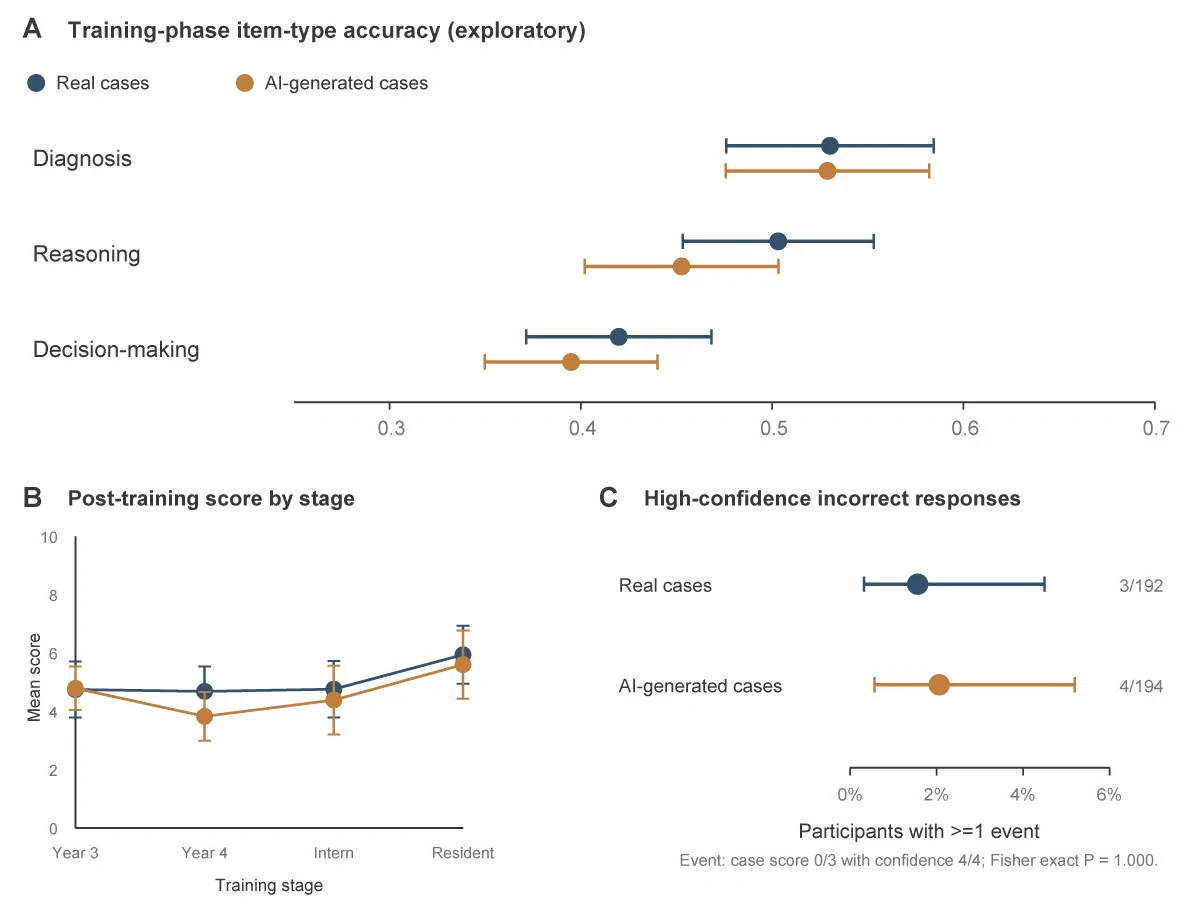
Exploratory and safety-related analyses. **(A)** Participant-level mean proportion correct for diagnosis-, reasoning-, and decision-making-labeled items among the 15 training-phase items. **(B)** Mean post-training test scores summarized by training stage; error bars indicate descriptive 95% confidence intervals. **(C)** Proportion of participants with at least one high-confidence completely incorrect response, defined as a per-case score of 0/3 with a confidence rating of 4/4. Error bars indicate exact 95% confidence intervals; Fisher’s exact test P = 1.000.

The exploratory analysis of training-phase item types found no significant between-group differences for diagnosis-, reasoning-, or decision-making-labeled items (Figure 3A). Post-training test results by training stage are shown in Figure 3B. In an exploratory post hoc analysis, 3 of 192 participants (1.6%) in the real-case group and 4 of 194 participants (2.1%) in the AI-generated case group had at least one high-confidence completely incorrect response; the risk difference was 0.5 percentage points (95% CI, –2.2 to 3.2 percentage points; P = 1.000; Figure 3C).

## 4. Discussion

In this trial, mean immediate post-training test scores were similar in the AI-generated and real-case groups (4.61 vs 4.95 points; mean difference, –0.335 points; 95% CI, –1.006 to 0.337), with no statistically significant between-group difference. However, because the lower bound of the confidence interval was below the prespecified non-inferiority margin of –0.5 points, non-inferiority was not demonstrated. A non-inferiority trial is designed to determine whether a new option is not appreciably worse than the comparator, not to prove equivalence [18]. Our findings therefore neither support the optimistic conclusion that AI-generated and real cases are interchangeable nor provide evidence that AI-generated cases are inferior. Rather, they indicate that, given the sample size and measurement precision of this study, the comparability of the two materials in immediate educational performance remains unresolved. The absence of significant between-group differences in training-phase performance, learning efficiency, perceived mental effort, satisfaction, and attitudes toward AI suggests that expert-reviewed AI-generated cases can support the short-term, online, text-based case tasks used here, but this does not demonstrate that they can replace real-case-derived teaching materials.

From the perspective of clinical reasoning education, the narrative complexity, atypical information, and conflicting cues carried by real cases might be expected to prompt deeper information filtering and hypothesis testing [19], and patient stories and illness scripts may help learners organize knowledge and develop diagnostic reasoning [20]. By contrast, LLM-generated cases tend to be more logically coherent and structured, which may help novices build frameworks but may also reduce exposure to authentic clinical uncertainty [10,21]. Our data did not support this theoretical expectation: the groups did not differ on diagnosis-, reasoning-, or decision-making-labeled training items. One plausible explanation lies in the measurement instrument. Clinical reasoning involves multiple processes, including information gathering, problem representation, hypothesis generation, differential diagnosis, and management decisions [22], whereas single-best-answer multiple-choice items, although convenient for standardization and between-group comparison, have limited ability to capture how learners form diagnoses, revise hypotheses, or handle uncertainty [23]. Thus, diagnosis, reasoning, and decision-making in this study should be understood as item-type labels rather than validated subscales of ability. Whether narrative complexity translates into a reasoning advantage should be tested with open-ended responses, completeness of the differential diagnosis, or performance-based assessment.

Evidence on authenticity also requires cautious interpretation. Although some AI-generated cases were judged as real, real cases were identified correctly more often, indicating that case-source masking was not entirely successful and that AI-generated cases cannot be assumed to have the same fidelity as real cases. This concern was most evident for Level 3 acute and critical cases, for which AI-generated cases received significantly lower realism ratings. This finding is consistent with prior observations that LLMs often perform well on standardized, structured tasks but remain limited in open-ended complex scenarios, flexible reasoning, and the handling of clinical detail [21]. Expert supervision was therefore not an optional step in our study [24]. Three senior experts reviewed each case pair for diagnosis, key cues, complexity, and educational suitability, and only unanimously endorsed pairs were included. Prior work has repeatedly noted that although AI can assist in generating cases and test items, the quality, clinical relevance, and reasonableness of the answers still require professional evaluation [25,26,27]. Human-AI collaboration, however, is not automatically beneficial; its effect depends on task design, the mode of supervision, user training, and how humans judge and integrate AI output [28]. Our findings therefore support positioning AI-generated cases as teacher-supervised case-development tools rather than ready-made substitutes for teachers or real cases.

The potential value of AI-generated cases for privacy and scalability is important, but the related claims should be narrowly framed [7]. The AI-generated cases in this study used matched, fully de-identified real cases as input and therefore were not synthetic cases independent of real patient data. Although this process reduced learners’ direct exposure to original records, it should not be interpreted as fully avoiding privacy-disclosure, re-identification, or data-governance risks. Prior work shows that identification risk persists when original records participate in data generation [29]. Access control over the original data, control of the model-use environment, documentation of the generation process, and expert review therefore remain indispensable. Under such governance, AI-generated cases can support standardization, rapid updating, and large-scale production of teaching materials, and can provide a supplementary option when case resources are scarce or materials must be tailored to specific learning objectives [9]. However, this study did not directly evaluate privacy-protection effectiveness, cross-institutional sharing, or large-scale generation. Therefore, its conclusions should not be extrapolated to AI-generated cases as a validated privacy-enhancing technology, nor should they be taken to show that AI-generated cases can replace real-world patient data [7].

### Limitations

This study has several limitations. First, it included only general surgery cases, so the conclusions may not generalize to other diseases, specialties, learning settings, or AI content that has not undergone expert review. Second, the primary outcome was a 10-item immediate post-training test administered without a pre-training test, delayed follow-up, or performance-based assessment. Therefore, learning gain, knowledge retention, and complete clinical reasoning ability could not be quantified; high-quality assessment of clinical reasoning should be supported by adequate validity evidence [30]. Third, the observed standard deviation of scores was higher than anticipated in the sample-size plan, widening the non-inferiority interval. Moreover, the –0.5-point margin was not a validated minimal educationally important difference, and there is no uniform methodological standard for setting or interpreting non-inferiority margins in this context [31]. Fourth, comparability of the two materials on direct textual features such as text length, information density, readability, and narrative structure was not quantified, so it cannot be determined whether the observed differences arose from case source or mode of presentation. Fifth, the AI-generated cases were produced using the default settings of Google AI Studio without a fixed random seed, and model behavior can drift across versions and over time; the generation process is therefore not deterministically reproducible [32].

## 5. Conclusion

In this short-term, online, text-based case-learning setting, expert-reviewed AI-generated cases and real-case-derived teaching materials did not differ statistically in immediate post-training performance, but non-inferiority of AI-generated cases was not demonstrated against the prespecified margin. For medical educators, these findings support using AI-generated cases as low-stakes, teacher-supervised supplements rather than as substitutes for real-case teaching, clinical exposure, or high-stakes competency assessment. Future research should improve the authenticity of complex cases and evaluate educational value systematically using larger samples, assessments with adequate validity evidence, pre-training and long-term follow-up, a broader range of clinical topics, and multimodal cases.

## Additional File

The additional file for this article can be found as follows:

10.5334/pme.2535.s1 Supplementary Materials. Supplementary Material S1, Tables S1 to S4 and Figure S1.

## Data Availability

The data supporting the findings of this study are not openly available because of sensitivity considerations but may be obtained from the corresponding author upon reasonable request.
