# Supplementary material for: Pedagogical Efficacy of LLM-Generated Synthetic Data Versus Real-World Clinical Records: A Randomized Controlled Non-Inferiority Trial
Source: Perspect Med Educ. 2026 Sep 9;15(1):759–70. doi: 10.5334/pme.2535 (PMC13564930; doi:10.5334/pme.2535)
Supplement: Supplementary file 1 [file pme-15-1-2535-s1.pdf]

Supplementary Materials

Contents

Supplementary Material S1. Study Instrument: Questionnaire, AI-Generated Clinical Cases, Items, Answer Keys, and Teaching–Assessment Blueprint

Supplementary Table S1. Outcome definitions and scoring

Supplementary Table S2. Item analysis of the 10-item post-training assessment

Supplementary Table S3. Outcomes by prespecified case complexity

Supplementary Table S4. Indirect comparability of case materials between groups

Supplementary Figure S1. Primary non-inferiority analysis of the post-training test score

Supplementary Material S1. Study Instrument: Questionnaire, AI-Generated Clinical Cases, Items, Answer Keys, and Teaching–Assessment Blueprint

Clinical Reasoning Training and Assessment Instrument — General Surgery (Acute Abdomen)

This supplement presents the complete study instrument. The study was conducted in Chinese; the text below is an English translation provided for review. The AI-generated case texts are presented in full. The full text of the real-case (control-group) teaching materials is not disclosed owing to patient-privacy and data-governance requirements; the indirect comparability of the two sets of materials is reported in Supplementary Table S4.

Instrument flow and teaching–assessment alignment

The sequence experienced by each participant was: informed consent and baseline → automated 1:1 random assignment of case source → five training cases, each followed by three scored items (diagnosis, reasoning, management) and case-perception ratings, after which a worked explanation conveying the transferable principle was shown → a 10-item post-training test → an overall evaluation. The platform automatically recorded per-case reading and answering times.

Test design. Each post-training test item was anchored to a transferable principle taught in one of the training cases and assessed application of that principle in a new clinical scenario rather than recall of case facts. The 10 items comprised 7 near-transfer, 2 far-transfer, and 1 floor item. The item-to-case mapping is provided in Appendix A2.

Fixed order within each case: read the case → answer 3 items → rate answer confidence → rate realism, perceived difficulty, and source → view the worked explanation. The explanation was always shown last, after the ratings had been recorded.

Part 1 | Informed consent

This study compares the learning effects of real de-identified clinical cases and expert-reviewed AI-generated clinical cases in general-surgery clinical reasoning training. Participation is entirely voluntary, the questionnaire is anonymous, and data are used for academic analysis only. Participants may withdraw at any time without any effect on their studies or training evaluation.

Note: This supplement reproduces the original instrument used during the study. The term "AI-generated cases" is used throughout; it denotes teaching cases generated by Gemini 3.0 Pro using matched, fully de-identified real

cases as input and subsequently reviewed by three senior (full professor) general-surgery experts. Such cases still derive from real patient information and are not fully synthetic data independent of real data.

☐ I have read and understood the above and voluntarily agree to participate in this study.

Part 2 | Baseline characteristics

B1 [Participant ID] Automatically generated anonymous ID: ________________

B2 [Sex] Your sex is?

1 = Male 2 = Female

B3 [Training stage] Your current training stage is?

1 = Year 3 (undergraduate) 2 = Year 4 (undergraduate) 3 = Intern 4 = Resident

B4 [General-surgery rotation] Have you completed a general-surgery clinical rotation?

0 = No 1 = Yes

B5 [Baseline confidence] How confident are you in managing a patient with an acute abdomen independently?

1 = No confidence at all 2 = Insufficient confidence 3 = Neutral 4 = Fairly confident 5 = Very confident

B6 [Learning preference] Which way of learning do you usually prefer?

1 = Self-directed reading of textbooks/guidelines 2 = Case discussion or bedside teaching 3 = Video or online courses

Part 3 | Clinical reasoning training across five cases

The platform displayed either real de-identified cases or AI-generated cases according to random assignment. Across the two groups, cases were matched on clinical topic, complexity level, and item structure. The AI-generated case versions are reproduced below; the real-case texts are not disclosed. Each case has three items corresponding to the diagnosis, reasoning, and management dimensions.

Case 1 | Level 1 | Acute uncomplicated appendicitis

Demographics: Male, 24 years, university student.

Chief complaint: Migratory right-lower-quadrant pain for 12 hours.

History of present illness: Twelve hours ago the patient developed dull, persistent epigastric and peri-umbilical pain without an obvious trigger, accompanied by nausea and reduced appetite, without vomiting. About 6 hours ago the pain migrated to and localized in the right lower

quadrant (RLQ), becoming persistent and distending with paroxysmal exacerbation, worsened by walking and coughing. Since onset there have been no chills or fever, no diarrhea, no urinary frequency/urgency or gross hematuria, and normal bowel movements and flatus. Appetite is poor; weight unchanged.

Past medical history: Previously healthy; denies hypertension, diabetes, or other chronic disease; denies hepatitis, tuberculosis, or other infectious disease; denies prior abdominal surgery or trauma; denies food or drug allergies.

Physical examination: T 37.6°C, P 88/min, R 18/min, BP 122/76 mmHg. Alert, acutely ill appearance, prefers to flex the right lower limb. Heart and lungs unremarkable. Abdomen flat and soft, no visible peristalsis; fixed tenderness at McBurney's point with mild rebound, no guarding; Rovsing's sign positive, psoas sign negative, obturator sign negative; liver and spleen not palpable below the costal margin; bowel sounds 4/min.

Investigations: CBC: WBC 11.5×10^9/L (↑), neutrophils 78% (↑), Hb 145 g/L; CRP mildly elevated; urinalysis normal (no RBC/WBC), helping to exclude ureteral calculus and urinary tract infection; abdominal ultrasound shows a blind-ended tubular structure in the RLQ, 0.8 cm in diameter, with a slightly thickened wall and no obvious surrounding effusion.

A. Clinical decision items (1 point each; 3 points total)

Q1.1 [Diagnosis] The most likely diagnosis is?

A. Acute gastroenteritis B. Acute appendicitis C. Right ureteral calculus D. Mesenteric lymphadenitis

Q1.2 [Reasoning] Which finding best supports this diagnosis?

A. Absence of fever B. Pain migrating from the epigastrium and localizing to the RLQ with McBurney's-point tenderness C. Normal urinalysis D. No obvious peritoneal effusion

Q1.3 [Decision-making] The most appropriate management is?

A. Discharge after antispasmodic/analgesic only B. Enema for constipation C. Complete preoperative assessment and perform appendectomy D. Long-term oral acid suppression

B. Confidence and case-perception ratings

C1.1 [Confidence] Overall, how confident are you that your answers to the three items are correct?

1 = No confidence / mainly guessing; 2 = Low confidence; 3 = Fairly confident; 4 = Very confident

C1.2 [Realism] How much does this case resemble a real clinical record?

1 = Not at all realistic; 2 = Somewhat unrealistic; 3 = Neutral; 4 = Fairly realistic; 5 = Very realistic

C1.3 [Perceived difficulty] How difficult do you consider the diagnosis and management of this case?

1 = Easy; 2 = Moderate; 3 = Difficult

C1.4 [Case source judgment] What do you think is the source of this case?

1 = Real case; 2 = AI-generated case; 3 = Cannot determine

Reference answers and transferable principles (Case 1)

Reference answers: Q1.1 → B; Q1.2 → B; Q1.3 → C.

Transferable principle ① (Diagnosis): "Migratory RLQ pain + localized peritoneal signs (fixed McBurney's-point tenderness)" is the core recognition pattern of acute appendicitis; normal urinalysis helps exclude ureteral calculus / UTI.

Transferable principle ② (Decision-making): After diagnosis, proceed to preoperative assessment and appendectomy rather than symptomatic analgesia and discharge. (Assessed downstream in items PT1 and PT2.)

Note: The worked explanation was shown to participants only after they had submitted their answers and completed the confidence and perception ratings for the case. It was instructional content and was not recorded as a study variable.

Case 2 | Level 2 | Incarcerated inguinal hernia with mechanical bowel obstruction

Demographics: Male, 68 years, retired worker.

Chief complaint: Painful, irreducible right inguinal bulge for 6 hours.

History of present illness: A reducible right inguinal mass for 2 years, reducible when supine or by manual pressure. Six hours ago, after vigorous coughing, the mass suddenly enlarged and protruded with severe distending pain and could not be reduced when supine or by manual maneuver. This was followed by abdominal distension, paroxysmal colicky pain, two episodes of vomiting gastric contents, and cessation of flatus and defecation. No hematemesis or melena; no obvious fever.

Past medical history: Chronic bronchitis with long-standing cough (a cause of raised intra-abdominal pressure); hypertension for 8 years on amlodipine, well controlled; denies prior abdominal surgery (no basis for adhesive obstruction); denies diabetes.

Physical examination: T 37.2°C, P 96/min, R 20/min, BP 132/80 mmHg. Alert, in pain. Abdomen slightly distended with visible intestinal contours and peristaltic waves; mild generalized tenderness, most marked in the RLQ; a 5×4 cm pear-shaped mass extending from the right groin to the scrotum, firm, tense, markedly tender, irreducible, with negative transillumination; hyperactive bowel sounds with an audible succussion splash and high-pitched metallic tinkles.

Investigations: Upright abdominal X-ray shows multiple step-ladder air-fluid levels in the mid-lower abdomen, consistent with mechanical bowel obstruction; CBC: WBC 12.8×10^9/L (↑), neutrophils 80%; blood lactate 2.6 mmol/L (↑, mildly elevated — alert to bowel ischemia).

A. Clinical decision items (1 point each; 3 points total)

Q2.1 [Diagnosis] The most likely diagnosis is?

A. Right hydrocele B. Right incarcerated inguinal hernia with mechanical bowel obstruction C. Inguinal lymphadenitis D. Simple constipation

Q2.2 [Reasoning] The progression most to be vigilant for at this point is?

A. Gastroesophageal reflux B. Strangulation of hernia contents and bowel necrosis C. Acute pancreatitis D. Urinary tract infection

Q2.3 [Decision-making] The most appropriate principle of management is?

A. Repeated forceful manual reduction then observation B. Laxatives only C. Defer to elective outpatient management D. Emergency surgical exploration after rapid assessment and resuscitation

B. Confidence and case-perception ratings

C2.1 [Confidence] Overall, how confident are you that your answers to the three items are correct?

1 = No confidence / mainly guessing; 2 = Low confidence; 3 = Fairly confident; 4 = Very confident

C2.2 [Realism] How much does this case resemble a real clinical record?

1 = Not at all realistic; 2 = Somewhat unrealistic; 3 = Neutral; 4 = Fairly realistic; 5 = Very realistic

C2.3 [Perceived difficulty] How difficult do you consider the diagnosis and management of this case?

1 = Easy; 2 = Moderate; 3 = Difficult

C2.4 [Case source judgment] What do you think is the source of this case?

1 = Real case; 2 = AI-generated case; 3 = Cannot determine

Reference answers and transferable principles (Case 2)

Reference answers: Q2.1 → B; Q2.2 → B; Q2.3 → D.

Transferable principle ① (Reasoning): In incarceration/obstruction, "persistent worsening severe pain + fever + peritoneal signs + elevated lactate/WBC" indicates bowel strangulation and ischemic necrosis.

Transferable principle ② (Decision-making): Ischemic bowel is a surgical emergency; repeated forceful reduction or mere observation is contraindicated, and emergency exploration after resuscitation is required. The principle "ischemic bowel = surgical emergency" transfers to other bowel-ischemia settings (PT3, PT4).

Note: The worked explanation was shown to participants only after they had submitted their answers and completed the confidence and perception ratings for the case. It was instructional content and was not recorded as a study variable.

Case 3 | Level 2 | Acute calculous cholecystitis

Demographics: Female, 45 years, schoolteacher.

Chief complaint: Persistent right-upper-quadrant pain for 1 day; fever with chills for 4 hours.

History of present illness: One day ago, after a fatty meal, persistent distending right-upper-quadrant (RUQ) pain with paroxysmal exacerbation, radiating to the right shoulder and back, with nausea and one episode of vomiting; 4 hours ago developed chills, rigors, and fever. No jaundice of skin or sclera; no clay-colored stools or tea-colored urine.

Past medical history: Gallstones for 3 years with intermittent RUQ dull pain, not regularly treated; G3P2, overweight (consistent with the "4F" profile); denies hepatitis.

Physical examination: T 38.2°C, P 96/min, R 20/min, BP 130/85 mmHg. Alert, no scleral icterus. Abdomen flat and soft; marked RUQ tenderness with guarding, Murphy's sign positive, hepatic percussion tenderness positive, shifting dullness negative, normal bowel sounds.

Investigations: CBC: WBC 14.2×10^9/L (↑), neutrophils 82% (↑); liver function: TBIL normal, ALT/AST normal; ultrasound shows an enlarged gallbladder (9×4 cm), wall thickness 0.4 cm with a double-wall sign, a 1.2 cm impacted stone in the neck, and a non-dilated common bile duct (inner diameter 0.5 cm).

A. Clinical decision items (1 point each; 3 points total)

Q3.1 [Diagnosis] The most likely diagnosis is?

A. Acute calculous cholecystitis B. Acute obstructive suppurative cholangitis C. Acute pancreatitis D. Perforated peptic ulcer

Q3.2 [Reasoning] Which best helps distinguish this from acute cholangitis?

A. Fever B. RUQ pain C. Absence of jaundice with normal total bilirubin and a non-dilated common bile duct D. Elevated WBC

Q3.3 [Decision-making] The appropriate treatment strategy is?

A. Discharge after oral analgesics only   B. Anti-infective and supportive therapy, and assessment for early laparoscopic cholecystectomy   C. Immediate common-bile-duct exploration and stone extraction   D. No treatment needed; regular follow-up

B. Confidence and case-perception ratings

C3.1 [Confidence] Overall, how confident are you that your answers to the three items are correct?

1 = No confidence / mainly guessing; 2 = Low confidence; 3 = Fairly confident; 4 = Very confident

C3.2 [Realism] How much does this case resemble a real clinical record?

1 = Not at all realistic; 2 = Somewhat unrealistic; 3 = Neutral; 4 = Fairly realistic; 5 = Very realistic

C3.3 [Perceived difficulty] How difficult do you consider the diagnosis and management of this case?

1 = Easy; 2 = Moderate; 3 = Difficult

C3.4 [Case source judgment] What do you think is the source of this case?

1 = Real case; 2 = AI-generated case; 3 = Cannot determine

Reference answers and transferable principles (Case 3)

Reference answers: Q3.1 → A; Q3.2 → C; Q3.3 → B.

Transferable principle ① (Diagnosis): Murphy's sign + thickened gallbladder wall with double-wall sign + impacted neck stone = acute calculous cholecystitis.

Transferable principle ② (Differentiating pivot): The presence/absence of jaundice, whether bilirubin is elevated, and whether the common bile duct is dilated are the core to distinguishing cholecystitis from cholangitis; all three are negative here, hence cholecystitis. This pivot transfers to "jaundice + biliary dilatation suggests cholangitis" (PT5, PT6).

Note: The worked explanation was shown to participants only after they had submitted their answers and completed the confidence and perception ratings for the case. It was instructional content and was not recorded as a study variable.

Case 4 | Level 3 | Perforated peptic ulcer with diffuse peritonitis

Demographics: Male, 35 years, company salesperson.

Chief complaint: Sudden knife-like severe epigastric pain rapidly spreading to the whole abdomen for 3 hours.

History of present illness: Three hours ago, sudden knife-like severe epigastric pain while at work, spreading to the whole abdomen within minutes, persistent, with profuse sweating and nausea,

without hematemesis or melena. History of recurrent epigastric discomfort, worse postprandially and at night; long-term self-medication with ibuprofen for low back pain over the past month. Since onset, the patient lies curled and still, refusing to move.

Past medical history: Chronic epigastric pain (suspected peptic ulcer), not formally treated; long-term NSAID use (ibuprofen); 10-year smoking history; denies prior surgery.

Physical examination: T 37.8°C, P 110/min, R 22/min, BP 100/60 mmHg. Acutely distressed, in a curled position. Abdominal respiration absent; whole-abdomen tenderness, rebound, and rigidity ("board-like" abdomen), most marked in the epigastrium; hepatic dullness reduced or absent, shifting dullness equivocally positive, bowel sounds absent.

Investigations: CBC: WBC 16.0×10^9/L (↑), neutrophils 88%; serum amylase mildly elevated (below the threshold for a pancreatitis diagnosis — helping to differentiate from acute pancreatitis); upright chest/abdominal X-ray shows a crescent of free gas under the right hemidiaphragm.

A. Clinical decision items (1 point each; 3 points total)

Q4.1 [Diagnosis] The most likely diagnosis is?

A. Acute simple gastritis B. Perforated peptic ulcer with diffuse peritonitis C. Acute cholecystitis D. Simple bowel obstruction

Q4.2 [Reasoning] The evidence that best supports perforation of a hollow viscus is?

A. Elevated WBC B. Heart rate 110/min C. Free subdiaphragmatic gas D. Prior ibuprofen use

Q4.3 [Decision-making] The primary management strategy is?

A. Proton-pump inhibitor only B. Resuscitation, anti-infective therapy, and emergency surgery as soon as possible C. Oral laxatives D. Await spontaneous resolution of pain

B. Confidence and case-perception ratings

C4.1 [Confidence] Overall, how confident are you that your answers to the three items are correct?

1 = No confidence / mainly guessing; 2 = Low confidence; 3 = Fairly confident; 4 = Very confident

C4.2 [Realism] How much does this case resemble a real clinical record?

1 = Not at all realistic; 2 = Somewhat unrealistic; 3 = Neutral; 4 = Fairly realistic; 5 = Very realistic

C4.3 [Perceived difficulty] How difficult do you consider the diagnosis and management of this case?

1 = Easy; 2 = Moderate; 3 = Difficult

C4.4 [Case source judgment] What do you think is the source of this case?

1 = Real case; 2 = AI-generated case; 3 = Cannot determine

Reference answers and transferable principles (Case 4)

Reference answers: Q4.1 → B; Q4.2 → C; Q4.3 → B.

Transferable principle ① (Diagnosis): Sudden severe pain + board-like abdomen + free subdiaphragmatic gas = perforation of a hollow viscus ("free gas" is the key reading and is not limited to ulcer perforation).

Transferable principle ② (Decision-making): Perforative peritonitis requires fluid resuscitation, broad-spectrum anti-infective therapy, and emergency surgery as soon as possible (PT7, PT8).

Note: The worked explanation was shown to participants only after they had submitted their answers and completed the confidence and perception ratings for the case. It was instructional content and was not recorded as a study variable.

Case 5 | Level 3 | Acute obstructive suppurative cholangitis with septic shock

Demographics: Female, 72 years, retired.

Chief complaint: RUQ pain, chills and high fever with altered consciousness for 1 day.

History of present illness: Twenty-four hours ago, persistent RUQ pain after eating, with recurrent chills and high fever (peak 39.5°C); subsequently progressive jaundice of skin and sclera with darkening urine; in the past 2 hours the patient developed somnolence, apathy, and incoherent responses, with markedly reduced urine output.

Past medical history: Common-bile-duct stones with prior cholecystectomy; hypertension and type 2 diabetes; denies hepatitis.

Physical examination: T 39.2°C, P 128/min, R 24/min, BP 80/50 mmHg. Confused (GCS 13), marked jaundice of skin and sclera, cold and clammy extremities. Abdomen flat and soft, RUQ tenderness with guarding, diminished bowel sounds.

Investigations: CBC: WBC 22.0×10^9/L (↑), neutrophils 92%, PLT 65×10^9/L (↓); TBIL 120 μmol/L (↑, predominantly direct), ALP/GGT elevated; Cr 150 μmol/L (↑); arterial blood gas pH 7.30, lactate 6.5 mmol/L (↑); upper-abdominal CT shows a markedly dilated common bile duct (1.5 cm) with an impacted distal stone.

A. Clinical decision items (1 point each; 3 points total)

Q5.1 [Diagnosis] The most likely current diagnosis is?

A. Acute viral hepatitis   B. Acute obstructive suppurative cholangitis with septic shock   C. Simple gallstones   D. Hepatic encephalopathy

Q5.2 [Reasoning] Abdominal pain, chills/high fever, jaundice, shock, and altered consciousness together are termed?

A. Murphy's sign B. Cullen's sign C. Charcot's triad D. Reynolds' pentad

Q5.3 [Decision-making] The best principle of management is?

A. Wait until antibiotics fully control infection before addressing the obstruction B. Antipyretics and analgesics only C. Urgent biliary decompression alongside aggressive resuscitation and anti-infective therapy D. ICU observation only

B. Confidence and case-perception ratings

C5.1 [Confidence] Overall, how confident are you that your answers to the three items are correct?

1 = No confidence / mainly guessing; 2 = Low confidence; 3 = Fairly confident; 4 = Very confident

C5.2 [Realism] How much does this case resemble a real clinical record?

1 = Not at all realistic; 2 = Somewhat unrealistic; 3 = Neutral; 4 = Fairly realistic; 5 = Very realistic

C5.3 [Perceived difficulty] How difficult do you consider the diagnosis and management of this case?

1 = Easy; 2 = Moderate; 3 = Difficult

C5.4 [Case source judgment] What do you think is the source of this case?

1 = Real case; 2 = AI-generated case; 3 = Cannot determine

Reference answers and transferable principles (Case 5)

Reference answers: Q5.1 → B; Q5.2 → D; Q5.3 → C.

Transferable principle ① (Reasoning): Charcot's triad (abdominal pain + chills/high fever + jaundice) further developing shock and altered consciousness constitutes Reynolds' pentad, indicating AOSC with septic shock.

Transferable principle ② (Decision-making, core): The key to obstructive suppurative infection is "source control" — urgent relief of the obstruction and drainage of the infection source alongside resuscitation and anti-infective therapy; one must not wait until infection is controlled before addressing the obstruction. This principle transfers to other obstructive sepsis (e.g., urosepsis; PT9, PT10).

Note: The worked explanation was shown to participants only after they had submitted their answers and completed the confidence and perception ratings for the case. It was instructional content and was not recorded as a study variable.

Part 4 | Unified post-training test (10 items, 10 points)

All participants completed the same items, 1 point each, closed-book and without reviewing the cases. Each item was adapted from a transferable principle of a training case and assessed application of the same logic in a new scenario. The item-to-case mapping is given in Appendix A2.

PT1 [Case 1 · Diagnosis · near transfer] A 28-year-old woman with peri-umbilical dull pain at onset; after about 6 hours the pain migrates to and localizes in the RLQ, with nausea and low-grade fever; localized tenderness at McBurney's point with mild rebound; normal urinalysis. The most likely diagnosis is?

A. Acute gastroenteritis B. Acute appendicitis C. Right ureteral calculus D. Acute pelvic inflammatory disease

PT2 [Case 1 · Reasoning · near transfer] To distinguish acute appendicitis requiring surgery from acute gastroenteritis, the most discriminating feature is?

A. Presence of nausea/vomiting B. Presence of diarrhea C. Whether the pain migrates and localizes to one site with localized peritoneal signs D. Presence of low-grade fever

PT3 [Case 2 · Reasoning · near transfer] A 70-year-old man with abdominal distension, vomiting, and cessation of flatus/defecation for 1 day, initially with paroxysmal colic; in the past 2 hours the pain has become persistent and severe, with fever, tachycardia, localized peritoneal signs, and elevated blood lactate. The greatest concern is?

A. Simple mechanical obstruction B. Functional ileus C. Acute gastroenteritis D. Bowel strangulation/necrosis

PT4 [Case 2 · Reasoning · far transfer] A 75-year-old patient with atrial fibrillation develops sudden severe abdominal pain markedly out of proportion to abdominal signs (early soft abdomen, mild tenderness), with marked restlessness and elevated lactate. The most appropriate principle is?

A. Observe and give symptomatic analgesia for 24 h B. Strongly suspect mesenteric ischemia; urgent assessment and early intervention C. Rehydrate as gastroenteritis and discharge D. Oral antibiotics only

PT5 [Case 3 · Diagnosis · floor item] A positive Murphy's sign is most commonly seen in?

A. Acute cholecystitis B. Acute appendicitis C. Perforated peptic ulcer D. Acute pancreatitis

PT6 [Case 3 · Reasoning · near transfer] A patient with RUQ pain and fever newly develops jaundice, markedly elevated total bilirubin, and a dilated common bile duct. Compared with simple acute cholecystitis, these findings most suggest?

A. Still simple acute cholecystitis B. Acute appendicitis C. Peptic ulcer D. Concurrent acute cholangitis / biliary obstruction

PT7 [Case 4 · Diagnosis · near transfer] An emergency patient with sudden whole-abdomen severe pain, a board-like abdomen, and absent hepatic dullness; an upright chest film shows bilateral free subdiaphragmatic gas. The most likely consideration is?

A. Solid-organ (liver/spleen) rupture B. Perforation of a hollow viscus C. Acute pancreatitis D. Mesenteric ischemia

PT8 [Case 4 · Decision-making · near transfer] Confirmed hollow-viscus perforation with diffuse peritonitis; the patient has tachycardia and low blood pressure. The most appropriate initial management is?

A. Conservative NPO plus PPI only B. Wait for contrast-enhanced CT before deciding C. Fluid resuscitation, broad-spectrum anti-infective therapy, and emergency surgery as soon as possible D. Oral laxatives

PT9 [Case 5 · Reasoning · near transfer] A patient with abdominal pain, chills/high fever, and jaundice then develops hypotensive shock and altered consciousness. This constellation (pentad) most suggests?

A. Acute simple cholecystitis B. Acute obstructive suppurative cholangitis (AOSC) C. Acute viral hepatitis D. Acute pancreatitis

PT10 [Case 5 · Decision-making · far transfer] A patient with ureteral-calculus urinary obstruction develops fever, chills, and hypotensive shock (urosepsis). Alongside fluid resuscitation and anti-infective therapy, the most critical principle is?

A. Relieve the obstruction only after infection is fully controlled B. ICU observation only
C. Urgent relief of the obstruction and drainage of the infection source (e.g., ureteral stent or percutaneous nephrostomy) D. Defer to elective management after vital signs fully stabilize

Part 5 | Overall evaluation

F1 [Perceived mental effort] How much mental effort did completing the case training and test require?

1 = Very low · 2 = Low · 3 = Fairly low · 4 = Slightly low · 5 = Moderate · 6 = Slightly high · 7 = Fairly high · 8 = High · 9 = Very high

This is a single-item perceived mental-effort rating (1–9); it is not the full NASA-TLX.

F2 [Satisfaction] Do you think this case exercise helped improve your clinical reasoning?

1 = Not helpful at all 2 = Slightly helpful 3 = Neutral 4 = Fairly helpful 5 = Very helpful

F3 [Attitude toward AI-assisted teaching] If expert-reviewed AI-generated cases were used for teaching in the future, your attitude would be?

1 = Support use 2 = Neutral or cautious support 3 = Oppose use

F4 [Open feedback] Do you have any further comments or suggestions about this case exercise? (optional)

____

____

Appendix A | Researcher coding, teaching–assessment blueprint, and scoring key (not shown to participants)

A1. Variable mapping and scoring

Module	Questionnaire item	Data variable	Coding / scoring
**Group assignment**	System 1:1 randomization	Group_Assign	0 = real, 1 = AI (participant level; five cases from the same source)
**Baseline**	B2–B6	Gender, Stage, Rotation_Exp, Pre_Conf, Learn_Pref	Coded by option
**Case complexity**	Fixed level per case	Cx_Level	C1=1, C2=2, C3=2, C4=3, C5=3 (constant)
**Case scoring**	Qx.1–Qx.3 per case	Cx_Q1–Q3_Score	Correct = 1, incorrect = 0
**Case total / confidence**	3 items per case / Cx.1	Cx_Total / Cx_Conf	0–3 / 1–4
**High-confidence completely incorrect response**	Combination of total and confidence	Cx_HighConfError	Cx_Total = 0 and Cx_Conf = 4 → 1
**Process time**	Reading page / answering page	Cx_Time_Read, Cx_Time_Ans	Automatically recorded (seconds)
**Case perception**	Cx.2–Cx.4	Cx_Realism, Cx_Diff_Perc, Cx_Source	1–5 / 1–3 / 1–3
**Training-phase performance score**	15 items across 5 cases	Intervention_Grand_Total	0–15
**Post-training test**	PT1–PT10	PT_Q1–PT_Q10	0/1 each
**Post-training total / pass**	PT sum / ≥6	PT_Total / PT_Pass	0–10 / ≥6 = 1
**Overall evaluation**	F1–F3	Mental_Effort, Satisfaction, AI_Attitude	1–9 / 1–5 / 1–3
**Open feedback**	F4	Feedback_Txt	Free text

A2. Teaching–assessment alignment blueprint (item → case / dimension / principle / transfer type / answer)

Post-training item	Corresponding case	Dimension	Transferable principle assessed	Transfer type	Answer
**PT1**	Case 1 (appendicitis)	Diagnosis	Recognition of migratory RLQ pain + localized peritoneal signs; urinalysis to exclude calculus	Near · new patient	B
**PT2**	Case 1	Reasoning	Differentiating pivot: surgical acute abdomen vs gastroenteritis	Near	C
**PT3**	Case 2 (incarceration/obstruction)	Reasoning	Early recognition of bowel strangulation/necrosis	Near · adhesive obstruction	D
**PT4**	Case 2	Reasoning / decision-making	Principle "ischemic bowel = surgical emergency"	Far · mesenteric ischemia	B
**PT5**	Case 3 (cholecystitis)	Diagnosis	Murphy's sign / recognition of cholecystitis	Floor item · recall	A
**PT6**	Case 3	Reasoning	Jaundice / biliary dilatation → cholangitis (differentiating pivot)	Near	D
**PT7**	Case 4 (perforation)	Diagnosis	Free subdiaphragmatic gas → hollow-viscus perforation	Near	B
**PT8**	Case 4	Decision-making	Initial management of perforative peritonitis	Near	C
**PT9**	Case 5 (AOSC)	Reasoning	Reynolds' pentad → AOSC	Near	B
**PT10**	Case 5	Decision-making	"Source control" principle for obstructive sepsis	Far · urosepsis	C

Structure: 5 cases × 2 items = 10 (1:2 mapping). Transfer distribution — 7 near, 2 far (PT4, PT10), 1 floor (PT5). Dimension distribution — 4 diagnosis, 4 reasoning, 2 decision-making, permitting exploratory analyses by item type.

A2.1 Case core teaching points → corresponding post-training items

Case 1: ① Diagnostic recognition of migratory RLQ pain + localized peritoneal signs → PT1; ② Differentiation and management of the surgical acute abdomen → PT2.

Case 2: ① Early recognition of bowel strangulation/ischemic necrosis → PT3; ② Principle "ischemic bowel = surgical emergency" (far transfer to mesenteric ischemia) → PT4.

Case 3: ① Diagnostic recognition of acute cholecystitis → PT5 (floor item); ② Jaundice / biliary dilatation as the pivot distinguishing cholecystitis from cholangitis → PT6.

Case 4: ① Free subdiaphragmatic gas → hollow-viscus perforation → PT7; ② Resuscitation + emergency-surgery decision for perforative peritonitis → PT8.

Case 5: ① Reynolds' pentad → recognition of AOSC → PT9; ② "Source control" principle for obstructive sepsis (far transfer to urosepsis) → PT10.

A3. Scoring key

Training cases: Case 1 — B, B, C; Case 2 — B, B, D; Case 3 — A, C, B; Case 4 — B, C, B; Case 5 — B, D, C.

Post-training test: PT1 B; PT2 C; PT3 D; PT4 B; PT5 A; PT6 D; PT7 B; PT8 C; PT9 B; PT10 C.

Post-training test answer distribution: A×1, B×4, C×3, D×2 (balanced to avoid fixed-position bias).

Supplementary Table S1. Outcome definitions and scoring

Domain	Outcome	Operational definition	Analysis note
Primary outcome	Post-training score	10 MCQs; total 0-10; higher scores indicate better performance.	Non-inferiority analysis; margin -0.5 points.
Secondary (learning)	Training-phase score	15 items across five cases (diagnosis, reasoning, decision; 1 point each); total 0-15.	Secondary outcome; Wilcoxon rank-sum test.
Secondary (learning)	Learning efficiency index	Training-phase score divided by total reading time across the five cases (minutes).	Secondary outcome; Welch t test.
Secondary (binary)	Post-training pass	Post-training score >=6/10.	Binary secondary outcome; Yates-corrected chi-square.
Process (per case)	Answer confidence	Single rating 1-4 after each case (case level).	Descriptive; used to define the safety-related event.
Perception (per case)	Perceived realism	Single rating 1-5 after each case.	Secondary/descriptive; analyzed overall and by complexity.
Perception (per case)	Perceived difficulty	Single rating 1-3 after each case.	Descriptive.
Perception (per case)	Case-source judgment	Judged real / judged AI / unsure, once after each of the five cases.	Descriptive task; not a blinding test.
Post-task	Perceived mental effort	Single-item rating 1-9 (not the full NASA-TLX).	Higher ratings indicate greater effort; Welch t test.
Post-task	Overall satisfaction	Single rating 1-5.	Secondary; category distribution compared.
Post-task	Attitude toward AI	Support / neutral / oppose (three categories).	Secondary; three-category distribution.
Safety-related event	High-confidence completely incorrect response	Case total 0/3 and confidence 4/4; participant-level occurrence of >=1 event.	Exploratory; Fisher exact test.

Definitions align with the revised manuscript and reviewer-response materials.

Supplementary Table S2. Item analysis of the 10-item post-training assessment

Item	Difficulty index	Corrected item-total correlation	Upper-lower discrimination	Alpha if item deleted
Post-training item 1	0.674	0.552	0.750	0.871
Post-training item 2	0.624	0.571	0.779	0.870
Post-training item 3	0.585	0.582	0.799	0.869
Post-training item 4	0.534	0.649	0.925	0.864
Post-training item 5	0.508	0.625	0.864	0.866
Post-training item 6	0.466	0.606	0.848	0.867
Post-training item 7	0.409	0.647	0.853	0.864
Post-training item 8	0.337	0.672	0.878	0.862
Post-training item 9	0.363	0.575	0.793	0.869
Post-training item 10	0.280	0.563	0.746	0.870

Overall Cronbach alpha = 0.879 (bootstrap 95% CI 0.861 to 0.895).

Supplementary Table S3. Outcomes by prespecified case complexity

Outcome	Level (cases)	Real, mean (SD)	AI, mean (SD)	Difference, AI - real (95% CI)	P
Perceived realism (1-5)	Level 1 (Case 1)	4.56 (0.61)	4.53 (0.59)	-0.03 (-0.15 to 0.09)	0.604
Perceived realism (1-5)	Level 2 (Cases 2-3)	4.59 (0.55)	4.64 (0.56)	0.05 (-0.02 to 0.13)	0.177
Perceived realism (1-5)	Level 3 (Cases 4-5)	4.56 (0.60)	4.02 (0.73)	-0.54 (-0.63 to -0.45)	<0.001
Reading time (s)	Level 1 (Case 1)	44.9 (6.1)	39.0 (6.3)	-5.89 (-7.12 to -4.65)	<0.001
Reading time (s)	Level 2 (Cases 2-3)	59.8 (6.4)	50.6 (6.6)	-9.19 (-10.17 to -8.22)	<0.001
Reading time (s)	Level 3 (Cases 4-5)	74.9 (6.8)	64.1 (6.6)	-10.80 (-11.80 to -9.79)	<0.001
Case score (0-3)	Level 1 (Case 1)	1.89 (1.14)	1.94 (1.11)	0.06 (-0.17 to 0.28)	0.613
Case score (0-3)	Level 2 (Cases 2-3)	1.48 (1.17)	1.47 (1.20)	-0.01 (-0.23 to 0.21)	0.946
Case score (0-3)	Level 3 (Cases 4-5)	1.21 (1.14)	0.99 (1.05)	-0.21 (-0.41 to -0.01)	0.038

Means (SD) are descriptive. Differences (AI - real), 95% CIs, and P values are from models with participant-clustered robust standard errors. Levels: Case 1 = Level 1; Cases 2-3 = Level 2; Cases 4-5 = Level 3.

Supplementary Table S4. Indirect comparability of case materials between groups

Metric	Adjusted difference, AI - real (95% CI)	P
Reading time (s)	-9.17 (-9.90 to -8.45)	<0.001
Answering time (s)	0.75 (-0.20 to 1.71)	0.123
Perceived realism (1-5)	-0.20 (-0.26 to -0.15)	<0.001
Perceived difficulty (1-3)	-0.01 (-0.05 to 0.02)	0.463
Training-case score (0-3)	-0.08 (-0.27 to 0.12)	0.442

Values are adjusted average differences (AI - real) with participant-clustered robust 95% CIs across the five matched case pairs. These are behavioral and perception proxies; direct text-feature metrics (e.g., word count, readability indices, counts of diagnostic cues or distractors) were not prespecified, and verbatim real-case texts were not available for direct textual comparison.

Supplementary Figure S1. Primary non-inferiority analysis of the post-training test score

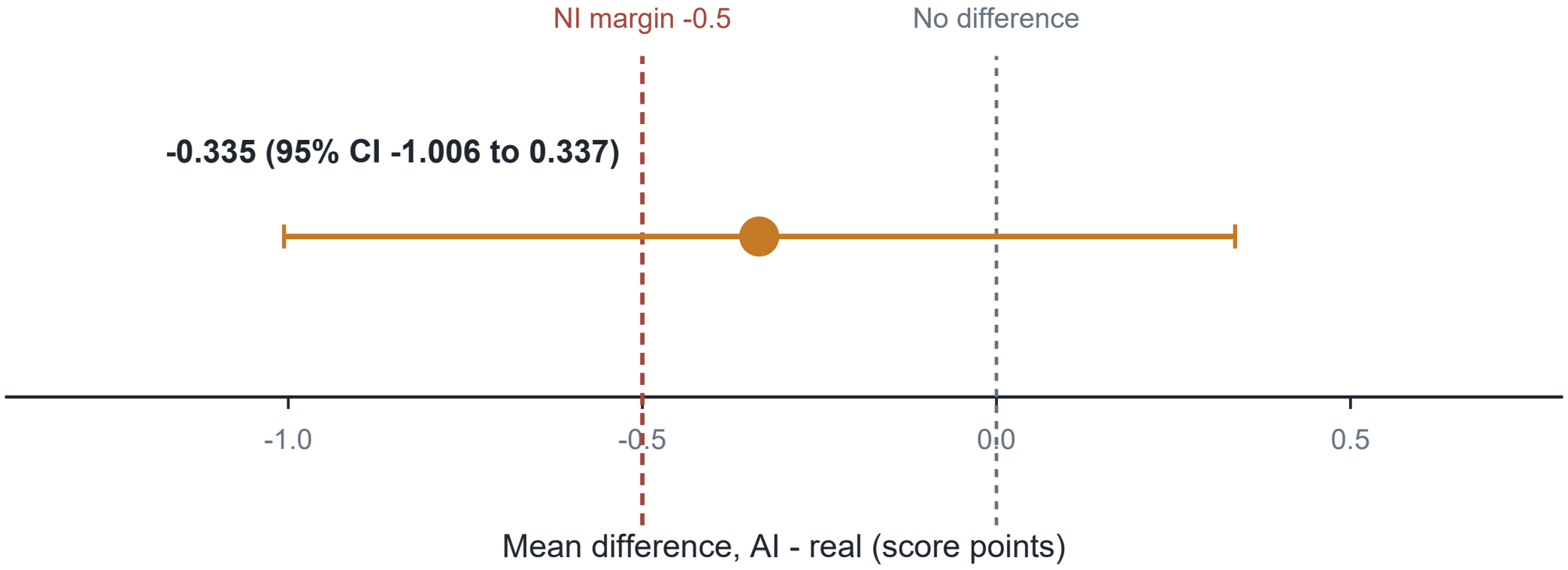

The confidence interval crosses the prespecified non-inferiority margin; non-inferiority was not demonstrated (one-sided P = .314).
